# Herbal Medicine in the Treatment of Non-Alcoholic Fatty Liver Diseases-Efficacy, Action Mechanism, and Clinical Application

**DOI:** 10.3389/fphar.2020.00601

**Published:** 2020-05-12

**Authors:** Yu Xu, Wei Guo, Cheng Zhang, Feiyu Chen, Hor Yue Tan, Sha Li, Ning Wang, Yibin Feng

**Affiliations:** School of Chinese Medicine, Li Ka Shing Faculty of Medicine, The University of Hong Kong, Hong Kong, Hong Kong

**Keywords:** non-alcoholic fatty liver disease, herb medicine, fatty acids, steatosis, inflammation

## Abstract

Non-alcoholic fatty liver disease (NAFLD) is a common chronic liver disease with high prevalence in the developed countries. NAFLD has been considered as one of the leading causes of cryptogenic cirrhosis and chronic liver disease. The individuals with obesity, insulin resistance and diabetes mellitus, hyperlipidaemia, and hypertension cardiovascular disease have a high risk to develop NAFLD. The related critical pathological events are associated with the development of NAFLD including insulin resistance, lipid metabolism dysfunction, oxidative stress, inflammation, apoptosis, and fibrosis. The development of NAFLD range from simple steatosis to non-alcoholic steatohepatitis (NASH). Hepatic steatosis is characterized by fat accumulation, which represents the early stage of NAFLD. Then, inflammation triggered by steatosis drives early NAFLD progression into NASH. Therefore, the amelioration of steatosis and inflammation is essential for NAFLD therapy. The herbal medicine have taken great effects on the improvement of steatosis and inflammation for treating NAFLD. It has been found out that these effects involved the multiple mechanisms underlying lipid metabolism and inflammation. In this review, we pay particular attention on herbal medicine treatment and make summary about the research of herbal medicine, including herb formula, herb extract and naturals compound on NAFLD. We make details about their protective effects, the mechanism of action involved in the amelioration steatosis and inflammation for NAFLD therapy as well as the clinical application.

## BACKGROUND

Non-alcoholic fatty liver disease (NAFLD) is a common chronic liver disease, and it is associated high metabolic risk of health problem such as dyslipidemia, insulin resistance, obesity and type II diabetic disease. In parallel with the prevalence of metabolic syndrome, NAFLD comprised of a spectrum of fatty liver that encompasses three typical pathological subtypes including liver steatosis, non-alcoholic steatohepatitis (NASH) and fibrosis. The least severe stage is simple liver steatosis induced by a largely build-up of fat in liver cell (Clapper et al., 2013). The progression of steatosis can be slowed or reversed by lifestyle modification and physical exercise. Otherwise, Non-Alcoholic Steatohepatitis (NASH), a severer form characterized by fat build-up, varying degrees of inflammation and ballooning degeneration of liver cells could be developed. More seriously, NASH continues to develop liver fibrosis and cirrhosis when the long term of liver injury leads to irreversible scarring of the liver (Bertot and Adams, 2019). As the late stage of fibrosis, Cirrhosis could irreversibly disrupt liver function and finally increased the risk of hepatocellular carcinoma development in patients.

Currently, there is no pharmacological agent that is being officially approved in NAFLD therapy. For one thing, the recommended intervention in NAFLD is lifestyle modification including energy intake restriction and physical activity enhancement. Lifestyle modification can reduce body weight and a moderate decrease of body weight could improve hepatic pathologic syndrome and decrease hepatic fat accumulation. For the other thing, some pharmacological interventions classified as antioxidants, insulin sensitizers and lipid-lowering drugs have been applied. For example, the antioxidant reagent such as Vitamin E has been suggested to treat non-diabetic patients with NASH as evidenced by a recent clinical trial (Sanyal et al., 2010); The use of lipid-lowering agents such as statin has been shown to reduce the risk of mortality or liver transplant in NAFLD patients (German et al., 2019); As sodium-glucose co-transporter 2 (SGLT2) inhibitors, Dapagliflozin and Canagliflozin could decrease hepatic lipid accumulation and significantly improve liver function (Arase et al., 2019). However, Most of these pharmacological agents are still at various stages of new drug development. For example, the natural farnesoid X receptor (FXR) agonist (obeticholic acid) and dual peroxisome proliferator-activated receptor α-δ (PPARα-δ) agonist (elafibranor), as well as glucagon-like peptide-1 antagonists were still investigated in phase IIA or IIB clinical trials (Sarwar et al., 2018). Meanwhile, several anti-diabetic medications such as pioglitazone (Brunner et al., 2019), metformin and thiazolidinediones have been applied for the pharmacologic management of NAFLD in clinical practice due to their ability to reverse insulin resistance. Although the advances in conventional medicine, herbal medicine are easily accessible and do not require artificial synthesis, thus herbal medicine seems highly attractive for the effective management of NAFLD. Herbal medicine, defined as whole medicinal plants and unpurified plant extracts with medical properties, has been traditionally used in different countries of the world to improve liver conditions. A special term Traditional Chinese Medicine (TCM) was particularly given to refer to herbal medicines that have been applied since the ancient time of China, though TCM might also include medicines with origins of animals and minerals. TCM includes various forms of herbal medicine that has been proved to take effects on treatment of NAFLD. In recent years, progress in drug development of NAFLD has been found major advances with herbal medicines which are regarded as abundant sources of natural bioactive chemicals that improve hepatic functions. In this paper, we have provided an overview of herbal medicine (including herbal formula, crude extract, and pure bioactive compound form medicinal plants) approaches which have demonstrated their ability to counteract NAFLD in human patients and animal models.

## The Effective Management of Herbal Medicine on NAFLD

### Herbal Medicine Improve Hepatic Lipid Metabolism

Overload lipid is the main initial reason that triggers hepatic steatosis. Excessive free fatty acids (FFAs) delivery from the adipose to the liver and result in the intrahepatic pool expansion of FFAs in the form of triglycerides. Fat accumulation evokes hepatic lipo-toxicity, which induces liver cells to release pro-inflammatory cytokines, trigger oxidative stress and hepatic stellate cell activation, ultimately lead to hepatic inflammatory injury. Improvement of fatty acid metabolism is an effective measure for in treating NAFLD, and the efficacy of herbal medicine targeting fatty acid metabolism has examined in both preclinical and clinical research of NAFLD. Firstly, The beneficial effects of herbal medicine on patients with liver dyslipidemia can improve lipid metabolic parameters such as decreasing the levels of triglycerides (TG), total cholesterol (TC) and low-density lipoprotein (LDL-C), alanine aminotransferase (ALT), aspartate aminotransferase (AST), as well as increasing the production of high-density lipoprotein (HDL-C). The interference of herbal medicine on dyslipidemia has been proved to be related to the regulation of fatty acid production or consumption. It has been found out that many herbal medicine (including herbal formula, crude extract and pure bioactive compound form medicinal plants) depressed the hepatic lipogenesis *via* reducing the expression of the key transcriptional factors and lipogenic enzymes such as Sterol Regulatory Element Binding Protein 1c (SREBP-1c), Peroxisome-Proliferator-Activated Receptor γ (PPAR-γ), Acetyl-CoA Carboxylase (ACC), Fatty Acid Synthase (FAS) and SCD1. For example, Gypenosides (extracted from *Gynostemma pentaphyllum*) (Li et al., 2017a), the chloroform fraction of *Cyclocarya paliurus* (Lin et al., 2016), total alkaloids extracted from *Rubus aleaefolius* Poir. (Li et al., 2014b), *Lonicera caerulea* L. extract (Park et al., 2019) and the crude extract from the peels of *Citrus aurantium* L. *(Rutaceae*) (Han et al., 2019) effectively attenuates high fat diet (HFD) induced triglyceride accumulation *via* reducing the high production of SREBP-1c, PPAR-γ, FAS, and ACC.

#### AMPK Pathway Involves in Herbal Medicine Modulation of Hepatic Lipogenesis and β-Oxidation

Adenosine monophosphate-activated Protein Kinase (AMPK) is a key energy sensor of intracellular energy metabolism, which could cause the reduction of cellular triglyceride and cholesterol production. The activation of AMPK phosphorylation could attenuate free fatty acid-regulated *de novo* lipogenesis genes and hepatic lipid accumulation. AMPK phosphorylation have been mentioned frequently in hepatic lipid metabolism to be activated in response to many herbal medicine such as BaiHuJia RenShen Decoction (Liu et al., 2015a), Qushi Huayu Decoction (Feng et al., 2013), *Lonicera caerulea* L. extract (Park et al., 2019), nobiletin (a polymethoxylated flavonoid derived from citrus fruits) (Yuk et al., 2018), ginsenoside Rb1 (Shen et al., 2013), betulinic acid (Kim et al., 2019b), and berberine (Zhu et al., 2019). Sophocarpine (derived from foxtail-like sophora herb and seed) influences adipocytokine production *via* AMPK signaling in NASH rats (Song et al., 2013), and salvianolic acid B (isolated from *Salvia miltiorrhiza* Bge.) reduces dyslipidemia and hyperglycemia *via* AMPK activation (Huang et al., 2016). It has been reported that some herbal medicine such as *Lonicera caerulea* L. extract (Park et al., 2019) and methanolic extract of *Alisma orientalis* (Hong et al., 2006) increases fatty acid β-oxidation *via* activating lipid antioxidant enzymes such as Carnitine Palmitoyltransferase-1 (CPT-1) and lessening peroxidation. This beneficial effects of herbal medicine on β-oxidation involved the activation of AMPK/PPAR-α and its downstream pathway. For example, the methanolic extract of *Alisma orientalis* (Hong et al., 2006), the ethanol extract of *Leonurus japonicus* Houtt (Lee et al., 2017), *Lycopus lucidus* Turcz. ex Benth (Lee et al., 2019) and Hugan Qingzhi formula (Yin et al., 2014) increases hepatic β-oxidation *via* upregulation of the phosphorylated AMPK and PPARα expression (Cao et al., 2016; Lee et al., 2017). AMPK activation in hepatic lipid β-oxidation also requires the activity of silent information regulator 1 (SIRT1), which interferes with PPARs activation. Silibinin shows its potential natural antioxidant effects on restoration of NAD^+^ levels *via* AMPK/SIRT1 pathway. Licochalcone A (isolated from *Glycyrrhiza uralensis*) significantly induces the AMPK/SIRT-1 pathway to inhibit hepatic lipogenesis synthesis and improve β-oxidation (Liou et al., 2019). Dioscin mediated SIRT1/AMPK signal pathway and LXRα action (Cheng et al., 2018) to modulate the expression of SREBP-1c, CPT-1, FAS, SCD, FoxO1, and ATGL(Yao et al., 2018). Ursolic acid has been treated as a novel Liver X receptor α (LXRα) antagonist and Ursolic acid stimulated AMPK phosphorylation to inhibit steroid receptor coactivator-1 (SRC-1) recruitment and promote small heterodimer partner-interacting leucine zipper protein to the SREBP-1c promoter region (Lin et al., 2018). Thus, AMPK action activated by herbal medicine involves in *de novo* lipid synthesis associated with the suppression of SREBP-1c, FAS, ACC, and SCD-1expression, and increase β-oxidation defense that improves hepatic fatty acids efflux *via* the modulation of CPT-1 and PPARα production.

#### Oxidative Stress Action Involves in Herbal Medicine Modulation of Lipid Metabolism

Oxidative stress reflected an imbalance between the reactive species production and antioxidant defense, which can lead to liver damage in the progression of NAFLD. The lipid metabolic disorder influences the production of reactive oxygen species (ROS), specifically, fatty acid β-oxidation seems to generate more ROS in NAFLD. The lipid lowering effect of herbal medicine shows its correlation with anti-oxidative stress action. For example, Bangpungtongseong-san attenuates the transcriptional response of oxidative phosphorylation (OXPHOS) in NAFLD liver (Choi J. Y. et al., 2019). Korea red ginseng shows anti-oxidant activity to improve hepatic lipid profiles in fatty rat (Hong et al., 2013). The ethyl acetate extract of *Aristolochia manshuriensis* Kom suppresses hepatic oxidative stress *via* improving the SOD, GR and GPx enzymes, subsequently increases hepatic lipid peroxidation of CYPE21 to promote hepatic lipolysis (Kwak et al., 2016). LiGanShiLiuBaWei San can significantly promote fatty acid oxidation *via* activation of PPARα and PPARβ, and reduce oxidative stress *via* the inhibition of iNOS production (Jiang et al., 2015). The down-regulation of hepatic HO-1, NF-E2-related factor 2 (Nrf2), and SOD2 as well as up-regulation KEAP1 were detected in the NAFLD models, and the expression of these oxidative stress markers can be all reversed by Dioscin (Liu et al., 2015b). The Nrf2 activation mediated by herbal medicine can improve NAFLD *via* inhibiting oxidative stress pathway. It has been reported that scutellarin (a flavonoid glycoside), swertiamarin (a secoiridoid glycoside) (Yang Y. et al., 2019) and gastrodin (isolated from *Gastrodia elata* Bl) enhance Nrf2-mediated antioxidant system *via* activating the mRNA and protein levels of PPARγ and its coactivator-1α, HO-1, GST, and NQO1 expressions (Ahmad et al., 2019), thus ameliorate NAFLD. Isochlorogenic acid B [extracted from *Laggera alata* (Asteraceae)] shows its protective effects on fibrosis in NASH by Nrf2 signaling pathway, and reverse the downregulation of miR-122 level and upregulation of hepatic HIF-1α expression to inhibit multiple profibrogenic factors (COL1α1, MCP-1, LOX, TGF-β1, and TIMP-1) (Liu et al., 2019c).

#### Mitochondria Function Involves in Herbal Modulation of Hepatic Lipid Metabolism

Mitochondria plays a specialized role in lipid metabolism and could contact lipid droplets *via* Mitochondria oxidation in liver. Mitochondria dysfunction contribute to the progression of NAFLD since it influences hepatic lipid metabolism, promote the generation of ROS, and lipid peroxidation. Previous research has proved that herbal medicine such as *cyclocarya paliurus*, *sida rhomboidea*.roxb, *punica granatum* L., resveratrol, mangosteen pericarp, epigallocatechin gallate, and shexiang baoxin pill, were shown to be effective on mitochondrial dysfunction during treating the NAFLD (Yang X. et al., 2019). More detail, Shizukaol D (extracted from *Chloranthus japonicas*) improved mitochondrial dysfunction, leading to hepatic AMPK-dependent lipid content reduction (Hu et al., 2013). Polygonati Rhizoma and *Polygonatum kingianum* promote mitochondrial β-oxidation *via* increasing the CPT-1 activity to block long-chain fatty acid enter mitochondria, and improve mitochondrial function *via* inhibiting HFD-induced excessive production of ROS and malondialdehyde (MDA) (Yang X. et al., 2019). Nobiletin (Qi et al., 2018) and diosgenin (Fang et al., 2019) could lead to the reduction of ROS level and restoration of mitochondrial membrane potential. This mitochondrial interference suppressed lipid peroxidation that involved the increase of vital scavenger levels of glutathione peroxidase (GSH-Px) and superoxide dismutase (SOD). Dioscin (isolated from *Polygonatum Zanlanscianense* Pamp) has been proved to increase the expression of SOD, GSH and GSH-Px, and decrease the production of iNOS, MDA and NO. Puerarin (extracted from Radix *Pueraria lobate*) evoked the activation of PARP-1/PI3K/AKT signaling that facilitated the transcripts (Acox1, MCAD, Cpt1 and Cox5a) in β-oxidation and reversed the decreased level of mitochondrial respiration complex I and II activities, further improved the fatty acid metabolism (Wang S. et al., 2019).

#### Bile Acid Synthesis Involves in Herbal Medicine Modulation of Lipid Metabolism

Bile acids are synthesized in the liver and act as biological detergent to metabolite lipids and cholesterol into the bile. Gypenosides (extracted from *Gynostemma pentaphyllum*) (Li et al., 2017a), palmatine and jatrorrhizine (extracted from Coptidis Rhizoma) promote bile acid synthesis to prevent NAFLD, which involved the expression of cholesterol 7α-hydroxylase A1 (CYP7A1). Punicalagin and pomegranate ellagic acid (isolated from *Punica granatum* L.) activate the CYP7A1/PPARγ signaling to promote hepatic diversion of cholesterol into bile acid (Ji et al., 2019). *Celastrus orbiculatus* Thunb. accelerated the hepatic cholesterol excretion in guinea pigs through suppressing oxidative stress *via* upregulation of the mRNA abundance of 3-hydroxy-3-methyl-glutaryl-CoA reductase (HMG-CoAR) and CYP7A1 (Zhang et al., 2013). Glycyrrhizin (extracted from Glycyrrhizae Radix Et Rhizoma) modulated serum bile acid metabolism in MCD diet-fed mice by restoring inflammation-mediated hepatic farnesoid X receptor (FXR) inhibition (Yan et al., 2018). The modulation of herbal medicine on lipid and cholesterol absorption and transport involved the bile acid regulation and shows its beneficial effects on treating NAFLD.

**Figure 1:**
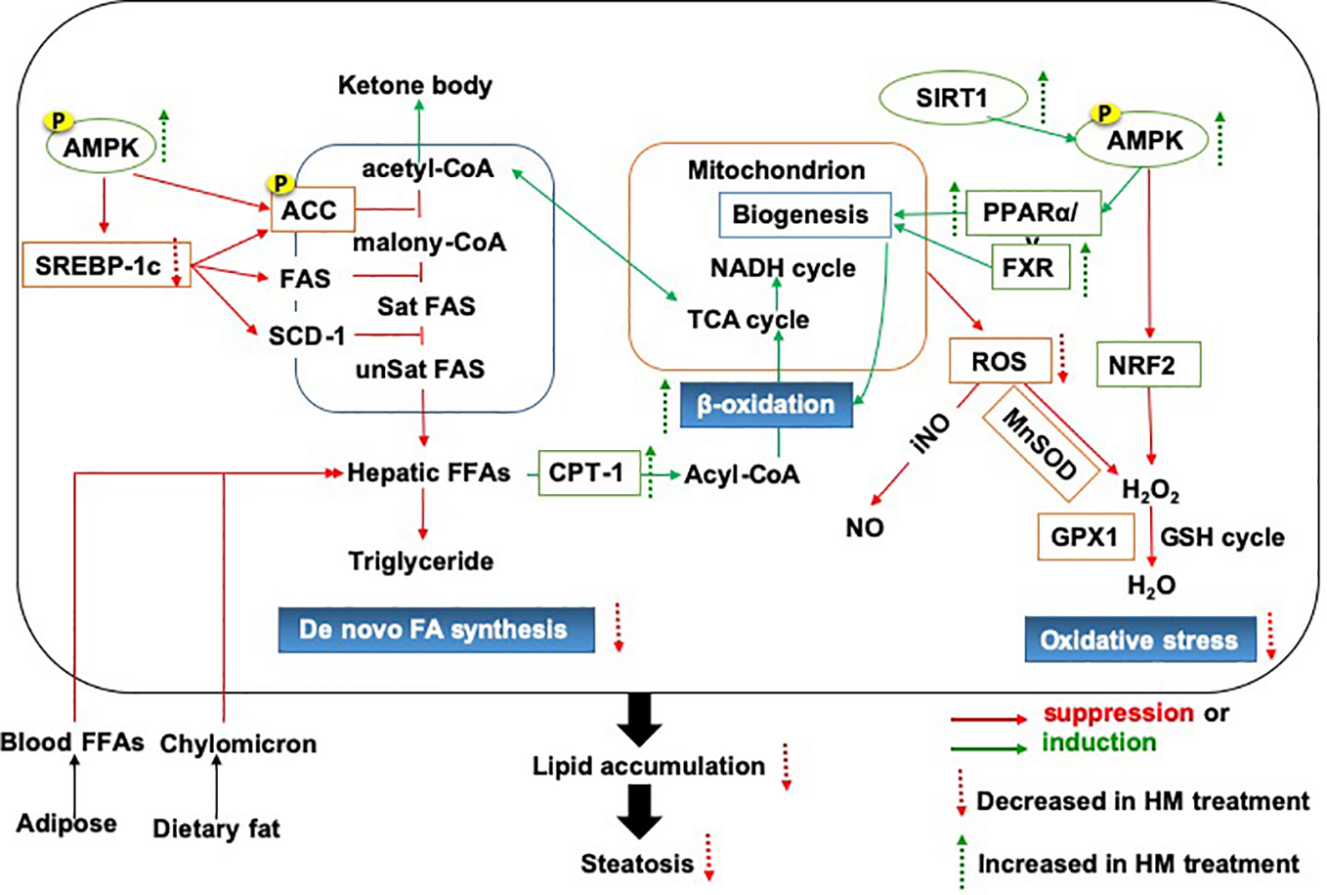
The lipid mechanistic insight into the anti-NAFLD effects of herbal medicine. NAFLD, Non-alcoholic fatty liver disease.

Above all, we provided an important lipid mechanistic insight into the anti-NAFLD effects of herbal medicine, which was shown in **Figure 1**. The commonly used herbal medicine inhibited fatty acid or diet-induced lipogenesis *via* SREBP-1c pathway and promoted the lipolysis focusing on fatty acid β-oxidation, which involved oxidative stress and mitochondrial function. The underlying mechanism of action might be achieved through the modulation of AMPK signaling pathway (Ding et al., 2019).

### Herbal Medicine Ameliorates the Hepatic Inflammation

**Figure 2:**
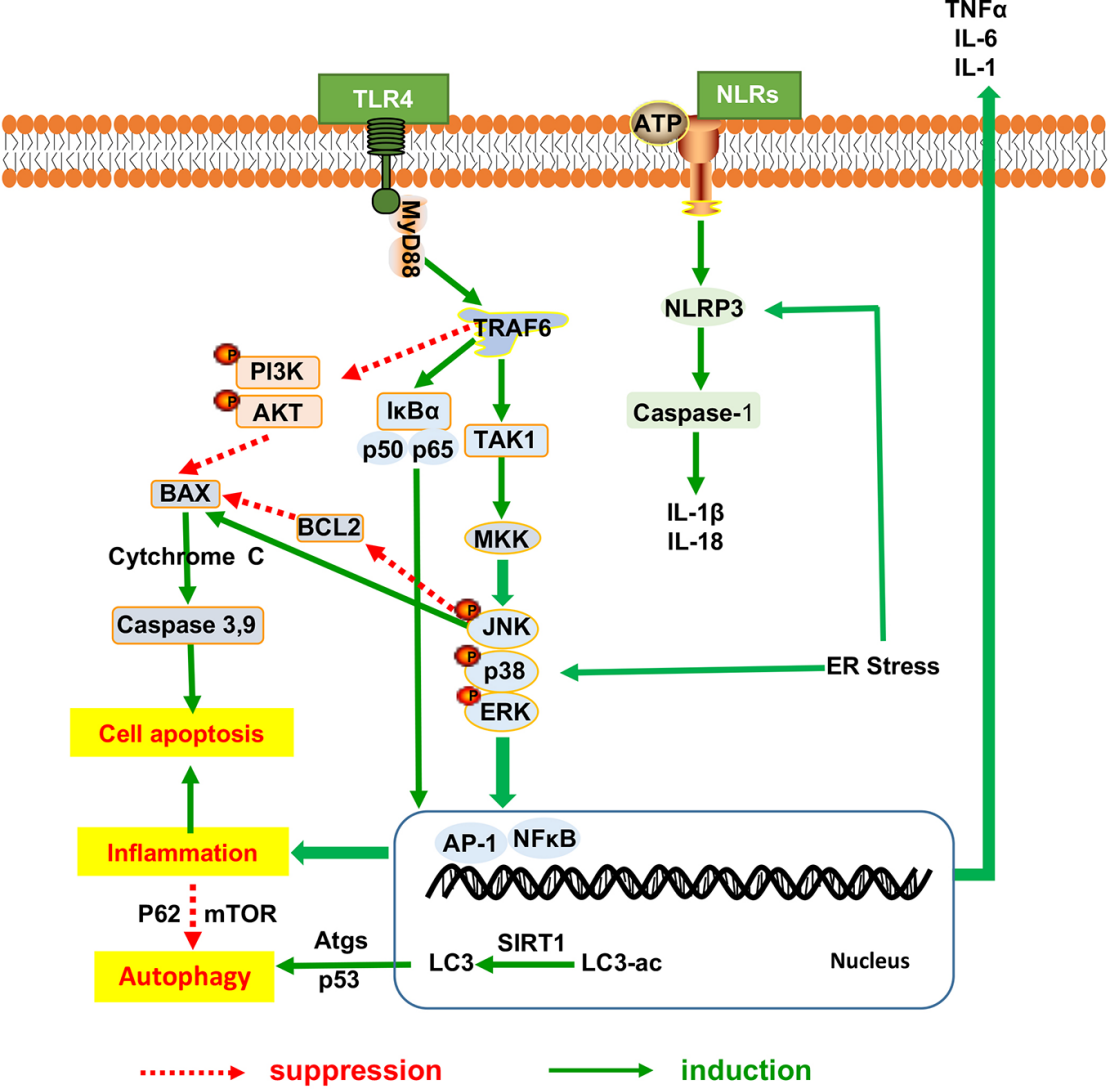
The inflammatory mechanistic insight into the anti-NAFLD effects of herbal medicine. NAFLD, Non-alcoholic fatty liver disease.

Hepatic inflammation promotes lipid deposition and redistribution from adipose to liver and drives liver inflammatory injury. Hepatic inflammation triggers the development of NAFLD from hepatic steatosis to steatohepatitis and fibrosis (Marra and Lotersztajn, 2013). Amelioration of heaptic inflammation is vital for NAFLD therapy. It has been indicated that herbal medicine has exerted the protective effects against the progression from hepatic steatosis to steatohepatitis and the underlying mechanism have been proved to be involved in inhibiting the inflammatory signaling pathway as shown in **Figure 2**, thereby regulating dyslipidemia and improving liver function in NAFLD. Many herbal medicine (including herbal formula, crude extract and pure bioactive compound form medicinal plants) possessed anti-inflammatory properties for slowing down the NAFLD progression, such as Sinai san dection (Zhang et al., 2005), Hugan Qingzhi tablet (Tang et al., 2015), betulinic acid (Kim et al., 2019b), *Alisma orientalis* (Choi E. et al., 2019), gastrodin (Ahmad et al., 2019), the peel extract of *Citrus aurantium L*. (Rutaceae) (Han et al., 2019) and swertiamarin (Yang Y. et al., 2019), and all of these medicine reduces the expression levels of hepatic inflammatory cytokines (TNFα, IL-6, and IL1β). Baicalin (extracted from *Scutellaria baicalensis* Georgi) (Zhang J. et al., 2018) and aqueous extract of *Salvia miltiorrhiza* Bunge (Hong et al., 2017) show the anti-inflammatory action, further improve liver fibrosis by inhibition of α-SMA, Col1A1, and TGF-β1 production.

#### NF-κB Pathway Involves in Herbal Medicine Modulation of Hepatic Inflammation

We found out that the NF-κB signaling mentioned frequently in the anti-inflammatory response of herbal medicine in treating NAFLD. Jianpi Huoxue formula (Feng et al., 2019), baicalin (Zhang J. et al., 2018) and *Lycium barbarum* polysaccharides showed ameliorative effects on hepatic inflammatory response involved with the reduction in Monocyte Chemotactic Protein-1 (MCP-1) expression, macrophage influx and hepatocyte apoptosis, partially owing to its power to suppress nuclear factor-κB (NF-κB) activation and the autophagic process of cleaved caspase-3 (Xiao et al., 2014; Zhai et al., 2015). Moreover, as a necessary prerequisite for triggering other inflammasome, the NF-κB signaling medicated the regulation of other inflammasome including NLRP3, the toll-like receptors and apoptosis mediators, all of which has been proved to be involved in the anti-inflammatory research of herbal medicine for NAFLD. Firstly, the palmitate acid-induced hepatocytes steatosis involved the activation of NLRP3 inflammasome and increased secretion of IL-1β and IL-18. And the NOD-, LRR- and pyrin domain-containing protein 3 (NLRP3) activation can be reversed by nobiletin and andrographolide (extracted from *Andrographis paniculata* (burm.f.)Nees) (Cabrera et al., 2017), as described by the downregulation of Caspase1, IL1β and IL18 expression *via* a NF-κB-dependent mechanism (Askari et al., 2014). Secondly, TLRs initiated signaling by binding to Myeloid Differentiation primary-response protein 88 (MyD88) triggers recruitment of TRAF6, TAK1, and MKK, which thereby activated the downstream signaling pathway of the extracellular signal-regulated kinase (ERK), p38MAPK, and c-jun N-terminal kinase (JNK). This activity leads to NF-κB nuclear translocation and the induction of gene transcription mediated by proinflammatory cytokines and chemokines. Toll-like receptors (TLRs) signaling pathways have been proved to participate in the anti-inflammatory response of herbal medicine in treating NAFLD. BuShenKangShuai tablet improved liver adiponectin resistance *via* inhibiting TLR4/NF-κB p65 signaling pathway, followed by the inhibition of TNF-α, IL-1β, MCP-1 and Vascular Vell Adhesion Molecule-1(VCAM-1), as well as the promotion of interleukin-10 and adiponectin production (Pang et al., 2019). Sparstolonin B (derived from *Sparganium stoloniferum* Buch.-Ham.) attenuated liver fibrosis through antagonizing TLR4 induced TGF-β signaling. Sparstolonin B augmented the hepatic TGFβ pseudo-receptor expression in mice, leading to downregulation of extracellular matrix deposition and hepatic stellate cell activation (Dattaroy et al., 2018). Moreover, Sparstolonin B obviously inhibited Kupffer cell activities as evidenced by the decrease in MCP-1 and CD68 levels with concomitant suppression of macrophage infiltration *via* blocking NADPH oxidase-driven TLR4 trafficking to the lipid rafts in NASH (Dattaroy et al., 2016). Dioscin rehabilitated inflammation (Liu et al., 2015b) that was associated with the decreased expression levels of p50, p65, and IκBα *via* regulation of MyD88-dependent TLR4 signaling pathway (Zhang E. et al., 2016; Yang L. et al., 2019). Garlic-derived S-allylmercaptocysteine mitigated NAFLD-induced inflammation *via* the restoration of the phosphorylated FFAs-dependent mitogen-activated protein kinases (MAPKs) and diminishment of the AP-1 and NF-κB activation (Xiao et al., 2013a). Berberine prevent NASH-derived hepatocellular carcinoma in mice *via* suppressing the phosphorylation of p38MAPK, ERK and COX2 expression (Luo et al., 2019).

#### Apoptosis Signaling Involves in Herbal Medicine Modulation of Hepatic Inflammation

The interference with the TLR mediated inflammatory response has proved to be involved into intracellular apoptosis signaling. These have been supported by the evidence that total aralosides from *aralia elata* (Miq) seem protected mice against HFD-induced cellular apoptosis, as suggested by TUNEL staining, and ameliorated NASH by inhibiting the NF-κB/IRE1α/JNK/IκB activation in ApoE^-/-^ mice (Luo et al., 2015); *Lycium barbarum* polysaccharides partially modulated hepatocyte apoptosis process *via* NF-κB/MAPK pathways, which has been proved to be related to the biological activity of l-arabinose and β-carotene in polysaccharides (Xiao et al., 2014). Resveratrol and Sparstolonin B exerted a promising role on the modulation of cell proliferation and apoptosis *via* TLR4/phosphatidylinositol 3-kinase- (PI3K-)/AKT signaling pathway (Dattaroy et al., 2016). *Psoralea corylifolia* L. inhibited NF-κB activation in the portal area to alleviate inflammatory cell infiltration and fibroplasia, further enhanced PI3K/Akt signaling to reduce hepatic superoxide anion expression, and NADPH oxidase activity as well as p47(phox) protein level and PKCα activation (Zhou et al., 2017). Since the apoptosis signaling involves in herbal medicine modulation of hepatic inflammation, herbal medicine reduces the apoptotic cytokines such as caspases and Bcl-2 family proteins expression in the progression of hepatic inflammation. For instance, dioscin increased Bcl-2 production and blocked the activation of Bak, Caspase-3/9, FasL, Fas, p53 proteins *via* reducing IRF9 production against apoptosis (Liu et al., 2015b; Shen et al., 2015; Zhang W. et al., 2016). Polygonati Rhizoma could notably remedy mitochondrial apoptosis and alleviate HFD-induced NAFLD *via* increasing the expression levels of caspase 3, caspase 9, and Bax, while decreasing the Bcl-2 level in hepatocytes and cytchrome C in mitochondria (Yang et al., 2019b). Furthermore, herbal medicine showed an effective role in inhibition of apoptotic cytokines, partially involved in the JNK-induced hepatocyte apoptosis for the treatment of NAFLD/NASH. Jianpi Huoxue formula (mainly consists of Atractylodis macrocephalae Rhizoma, Salvia miltiorrhizae Radix Et Rhizoma, Paeonia Radix Alba, Alismatis Rhizoma, and Schisandrae Chinensis Fructus) may have beneficial effects on MCD induced liver inflammation and apoptosis *via* the inhibition of the JNK phosphorylation and the activation of caspase 3 and 7 proteins (Feng et al., 2019). The ethyl acetate extract of *Aristolochia manshuriensis* Kom inhibited hepatic apoptosis *via* the suppression of ERK1/2 and JNK1/2 phosphorylation (Kwak et al., 2016); Silibinin (a flavonolignan from milk thistle) ameliorated various symptoms of NASH by activating death domain-like apoptosis regulator (CFLAR)-JNK pathway, and thereby modulated its downstream target genes to promote the fatty acid β-oxidation (PPARα, SREBP-1C, and PNPLA3), anti-oxidase action (CAT, HO-1, and GSH-Px) and inhibition of pro-oxidase action (NRF2, CYP4A, and CYP2E1) to ameliorate oxidative stress, as well as inflammatory response (Wang et al., 2017; Liu et al., 2018; Liu et al., 2019d). Therefore, activated hepatic apoptosis exerted a wide range of biological actions involved facilitating inflammation with oxidative stress modulation. And combined with the beneficial effects on liver steatosis, herbal medicine also relived the reliable growth in indicators of hepatocyte cytokine proinflammatory status, apoptosis, and fibrosis in the development of steatohepatitis.

#### Autophagy Involves in Herbal Medicine Modulation of Hepatic Inflammation

The lysosomal-mediated degradation process of autophagy is beneficial for removing the damaged cellular proteins and organelles, including mitochondria, peroxisomes, and endoplasmic reticulum (ER), thus the autophagy activation takes metabolic intermediates necessary for protein metabolism. And herbal medicine induced autophagy had been considered as an anti-inflammatory strategy to show the obvious effects on reducing the NAFLD progression. Resveratrol promoted the autophagy pathway to restore liver injury, which was linked with NF-κB activation (Ji et al., 2015); *Lycium barbarum* polysaccharides showed ameliorative effects on autophagic proteins (LC3II and Atg5), and deceased autophagic negative modulators (p62 and p-mTOR) *via* the NF-κB/MAPK pathways (Xiao et al., 2014). Herbal medicine rehabilitated inflammation, reduced apoptosis and enhanced autophagy through both extrinsic and intrinsic apoptotic pathways, thereby counteracting the effects of NAFLD. For example, Dioscin inhibits collagen synthesis through modulating the expressions of autophagic flux (P62 LC-I LC-II) (Xu et al., 2017). Administration of Akebia saponin D (extracts from *Akebia quinata*) resulted in the upregulation of autophagic flux (e.g., decreased P62 accumulation and increased the level of LC3-II expression) in the liver of ob/ob mice (Gong et al., 2016); Glycycoumarin (a coumarin compound isolated from Rhizoma Glycyrrhizae) inhibited hepatocyte lipo-apoptosis through reactivation of impaired autophagy and inactivation of GSK-3. In line with autophagy activation, mitochondrial apoptotic activation, and ER stress-mediated JNK pathway were also blocked by glycycoumarin (Zhang A. et al., 2016); Nobiletin (Yuk et al., 2018), ginsenoside Rb2 (extracted from *Panax Ginseng* C.A.Mey) and Tangshen formula (Wang Y. et al., 2019), could reverse the repression of autophagic pathways in AMPK-SIRT1 dependent manner (Huang et al., 2017). The AMPK/SIRT1 pathway mediated the activation of fork head box transcription factors that increased Atg proteins production, additionally, SIRT1 could combine with Atgs (Atg7 and Atg8) to form a molecular complex to deacetylate the core autophagy machinery. Resveratrol (a pharmacological SIRT1 activator) significantly prevented hepatocyte ballooning and steatosis, along with the changed levels of LC3-II, Beclin 1, and P62, as well as ER stress (Ding et al., 2017). It could be confirmed that resveratrol potentiated SIRT1 secretion and its deacetylase activity, which decreased p53 and increased autophagy. Further studies demonstrated that the effect of resveratrol on hepatic steatosis was achieved partially *via* the cAMP-PRKA-AMPK-SIRT1 autophagy pathway (Zhang et al., 2015). Meanwhile, mTOR, another famous negative autophagic regulator, was decreased after some herbal treatments, such as Garlic-derived S-allylmercaptocysteine enhanced the levels of autophagic markers expression in the liver, with a concomitant decrease the mTOR activity (Xiao et al., 2013b).

### Herbal Medicine Triggers Other Pathways in NAFLD

#### Endoplasmic Reticulum (ER) Stress

ER stress involved the increase of JNK and caspase-12 expression serves a primary role in the procession of NAFLD and pathogenesis to NASH. The pharmacological activation of FXR induced by betulinic acid alleviated the hepatic ER stress-mediated hepatic steatosis (Gu et al., 2019). The farnesoid X receptor (FXR) activation suppresses the expression of ER stress markers (PERK, EIF2α and ATF4) and CHOP signaling, thereby reducing hepatocellular ER stress. Betulinic acid could serve as an FXR agonist that effectively attenuates the pathogenesis of HFD and MCD induced NAFLD, and *Alisma orientalis* could restore the hepatocellular ER homeostasis by stimulating the FXR activation, particularly, Alisol A 24 (B 23)-acetate accounts for this action (Choi E. et al., 2019).

#### Insulin Signaling Pathway

Insulin-induced insulin receptor phosphorylation recruited the insulin receptor substrate-1 (IRS-1) and subsequently activated phosphoinositide 3-kinase (PI3K)/AKT pathway, leading to the activation of PKA and SGK-3β and ultimately encouraging glycogen and lipolysis synthesis, and subduing gluconeogenesis and lipogenesis (Kwon et al., 2018). Naringenin (Mulvihill et al., 2009), Ginsenoside Rb1(Yu et al., 2015) and *Leonurus japonicus* Houtt extract (Lee et al., 2017) prevented hyperinsulinemia, leading to the correction of NAFLD associated metabolic disturbance that was linked to glucose utilization and insulin sensitivity (Lee et al., 2017). It has been found that nobiletin (Kim et al., 2017) and ursolic acid (Li et al., 2014a) ameliorated insulin resistance and takes effects on amplifying glucose uptake through IRS-1/AKT stimulation (Yuk et al., 2018) in treatment of NAFLD; Jwa Kum Whan ameliorated insulin resistance and reduced hepatic triglyceride and cholesterol accumulation *via* significant triggered the phosphorylation of IRS-1 and PI3K (Lim et al., 2019). The potential underlying mechanism of Erchen decoction for the treatment of obesity, hyperlipidemia and fatty liver is to increase the CDKAL1 production, improve islet cell function and insulin level (Gao et al., 2015). A related study indicated that yangonin increased insulin sensitivity *via* increasing the expression of phosphorylated IRS-1 and IRS-2 (Dong et al., 2019).

### Herbal Medicine Induced Gut Microbiota Alteration in NAFLD

It has been suggested that gut dysbiosis in patients have different shift in the development of NAFLD. There is an inverse association between the presence of NAFLD and the abundance of *Bacteroidetes* in gut microbiota, indicating that the intestine microbiota takes a vital role in the progression of NAFLD (Mouzaki et al., 2013). Therefore, the modulation of intestine microbiota has become a potential therapeutic strategy for the treatment of NAFLD. Supplementation of prebiotic, probiotic compounds, or herbal medicine (Porras et al., 2017), have taken a modulatory effect on the intestine microbiota. Lingguizhugan decoction reduced hepatic steatosis and improved glycemic control through the modulation of gut microbiota (Liu et al., 2019b). Microbiome analysis revealed that herbal formula shenling baizhu powder promoted the relative percentage of short-chain fatty acid-producing microbiota, such as *Bifidobacterium* and *Anaerostipes* (Zhang Y. et al., 2018). Mechanistic studies found that herbal formula such as shenling baizhu powder alleviated hepatic steatosis and repaired colon mucosa *via* decreasing the expression level of endotoxin and inflammatory mediators (TNF-α, IL-1β) *via* the TLR4 pathway (Zhang Y. et al., 2018b). Diammonium glycyrrhizinate is a medicinal form of glycyrrhizic acid and has been proved to modify gut microbiota composition to decrease the intestinal low-grade inflammation and restore intestinal barrier function in NAFLD mice. Diammonium glycyrrhizinate reduced the abundance of the endotoxin-producing bacteria such as *Desulfovibrio* and elevates the level of probiotics such as *Lactobacillus* and *Proteobacteria*, as well as augmented the abundance of short-chain fatty acid (SCFA)-producing bacteria such as *Ruminococcaceae* and *Lachnospiraceae* to promote SCFA production (Li et al., 2018).

### Clinical Application of Herbal Medicine on NAFLD

In the clinical setting, clinical trials could confirm the experimental benefits of herbal medicine application in NAFLD patients. Clinical trials for NAFLD have been seen improvement with herbal medicine therapy. Randomized controlled trials comparing either herbal medicine alone or in combination with other interventions or pharmaceutical agents have been investigated and the results indicated that herbal medicine had a better effect on the normalization of AST and the disappearance of radiological steatosis in the treatment of NAFLD patients (Shi et al., 2012). For instance, the daily consumption of resveratrol plus with lifestyle modification for 12 weeks showed superior effect than lifestyle change alone. Meanwhile, after resveratrol supplementation, the decreased level of insulin resistance, ALT, AST, LDLC, TC, and TNF-α were showed in NAFLD patients (Berman et al., 2017). It is essential to prove the efficacy and safety of herb medicine in treating NAFLD, because the side-effects of herb medicine need to be confirmed and investigated further (Rahmani et al., 2016) in clinical application. Because some herbal medicines such as *Phyllanthus urinaria* L. that has been suggested as the hepatoprotective herb in animal studies showed unsatisfactory effect in improving NAFLD activity score in NASH patients (Wong et al., 2013). The development of Radom Control Trials for herbal medicine is important and essential for making validation about the efficacy of herbal medicine in treating NAFLD. We made summery about the clinical trials about the effective herbal medicine (including herbal formula, crude extract and pure bioactive compound form medicinal plants) that have proved to take positive effects on the biochemical and physiological features of NAFLD (Liu et al., 2013).

#### Herbal Formula Against NAFLD

According to Chinese Medicine theory and prescription principles, the herbal formula is developed with selection of appropriate medicinal plants and the dosage of each herb for treating patients with NAFLD. The meta-analysis of 62 randomized controlled trial was conducted to investigate the herbal medicine therapy for NAFLD. It indicated that 246 Chinese herbs have been found to be included and clinical applied for NAFLD with an average of ten species in each formulation. It was found out that herbal medicine take effective action on the therapy of NAFLD, and Crataegi Fructus (Shan-Zha) was the most common used herbal medicine for 321 times in 17,670 patients (Shi et al., 2012). Many herbal formulas have been reported to show anti-NAFLD function in clinical application. For example, Yinchenhao Decoction has been used in treatment of gallbladder and liver diseases for centuries and it was composed of *Artemisia capillaris* (Thunb), *Gardenia jasminoides* (Ellis), and *Rheum palmatum* (L) (Yao et al., 2016); Oral administration of Danning Tablet (composed of Rhei Radix Et Rhizoma, Polygoni Cuspidati Rhizoma Et Radix, dried green orange peel and dried old orange peel) for three month in 232 patients can improve the clinical symptoms of NAFLD (Fan, 2004); The results of meta-analysis showed that HuoXueHuaYu improved B ultrasonic level in patients with NAFLD. As to lipids, HuoXueHuaYu showed effective action on reduction of TC, TG, ALT, and AST levels (Cai et al., 2019); Bangpungtongseong-san is an ancient Chinese herbal medicine formula and has been clinically applied in Korea, Japan (Bofu-tsusho-san), and China (Fang feng tong sheng-san) for obesity and its associated metabolic syndrome (Kobayashi et al., 2017); Erchen Decoction is used for the treatment of obesity, hyperlipidemia, and fatty liver diseases. Seven randomized controlled trials with a total of 1951 participants were investigated for the effects of Erchen Decoction on patients with NAFLD. The meta-analysis results of Erchen Decoction investigation showed that patients receiving Erchen Decoction with conventional treatment showed more effective in clinical improvement of NAFLD compared with conventional treatment alone (Li et al., 2017b); The Dava AL-balgham (composed of *Nigella Sativa*, *Zataria Multiflora*, *Trachyspermum ammi*, *Pistacia lentiscus* have shown anti-inflammatory, anti-atherogenic, and anti-oxidant effects. A double blind randomized trial of Dava AL-balgham has been applied for 76 patients with fatty liver disease. After three month treatment, Dava AL-balgham could improve the serum level of liver enzymes in patients with fatty liver (Hormati et al., 2019).

#### Single Herb Against NAFLD

The meta-analysis was performed to confirm the efficiency and safety of *Salvia miltiorrhiza* Bunge (Danshen) in eight randomized controlled trials with 800 patients. It indicated that *Salvia miltiorrhiza* Bunge showed positive effects on the levels of ALT, AST, TC and TG, LDL, and liver/spleen computed tomography ratio in patients with NAFLD. Future randomized clinical trials of higher quality are still required to evaluate the efficacy and safety of *Salvia miltiorrhiza* Bunge in NAFLD (Peng et al., 2016). *Panax Ginseng* C.A.Mey has been often applied against multiple metabolic conditions, including hepato-steatosis, Korean Red Ginseng shows anti-inflammatory and anti-fatigue effects on 80 patients with NAFLD (Hong et al., 2016).

#### Pure Natural Compound Against NAFLD

Curcumin (a natural polyphenol from *Curcuma longa* L.) owed the lipid-modifying, anti-inflammatory and antioxidant properties. Panahi et al. reported a randomized placebo controlled trial of curcumin in 87 subjects with NAFLD (Panahi et al., 2017) and concluded that daily supplementation of curcumin for 8 weeks decreased liver lipid accumulation and the levels of AST and ALT in patients of NAFLD without any issues of tolerance (Liu et al., 2019a). Diammonium glycyrrhizinate, a medicinal form of glycyrrhizic acid possesses anti-inflammatory and antioxidant effects. It has been applied for the treatment and control of chronic hepatopathy including NAFLD(Li et al., 2018); Cinnamon (Blevins et al., 2007) showed the improvement of the serum glucose and lipid levels in people with non-insulin dependent type 2 diabetes mellitus (NCT00237640). Further studies with 50 patients NAFLD were tested to investigate whether Cinnamon exerts the insulin sensitizer action in NAFLD patients. The results showed that daily intake of Cinnamon (1.5 g) for 12 weeks has beneficial effects on lipid profile, insulin resistance, liver enzymes, and high-sensitivity C-reactive protein in NAFLD patients (Askari et al., 2014). Ginger possess strong antioxidant ability to reduce lipids peroxidation (Si et al., 2018). The Early Phase I clinical investigation of Ginger in treatment of NAFLD in patients with type 2 diabetes mellitus (NCT02289235) is in progress to test whether Ginger takes effects on the liver biomarkers (ALT, AST, and γ-glutamyl transpeptidase) and fatty liver score in fibro-scan. A multi-center, phase III, double-blind clinical trial reported that oral administration of silybin combined with phosphatidylcholine and vitamin E for 12 months can improve insulin resistance, liver enzymes, and liver histology (Loguercio et al., 2012). The clinical trial study of Silymarin (IRCT201202159018N1) was conducted on 64 patients with NASH and after 8 weeks treatment, the patients with NASH experience obvious fall in hepatic enzymes (Solhi et al., 2014).

#### Others

At present, the clinical trials recorded in U.S. National Library of Medicine mentioned the herbal medicine in treating NAFLD includes YiQiSanJu formula (NCT01677325, Phase I completed), Phyllanthus urinaria (NCT01210989, N/A completed), Zhenzhu Tiaozhi capsules (NCT03375580, N/A Recruiting), Fermented ginseng powder (NCT03260543, N/A completed), Trigonella Foenum-graecum Seed Extract (NCT02303314, Phase II, and III completed), *Zataria Multiflora* Boiss. (Shirazi’s thyme) (NCT02983669, N/A completed), and Ginger (NCT02535195, Phase II and III completed), as well as some bioactive natural compounds such as Berberine (NCT04049396, NCT03198572 Recruiting, Phase IV), Curcumin (NCT03864783, Recruiting), Silymarin (NCT02006498, Phase II completed and NCT02973295, Phase IV Recruiting), Resveratrol (NCT02030977, Phase II and III completed; NCT01464801, N/A completed; NCT01446276 N/A completed), Anthocyanin (NCT01940263, Early Phase I completed), Pioglitazone and Berberine (NCT00633282, phase II Completed), Anthocyanin (NCT01940263, phase II Completed) and Siliphos (NCT00443079, phase II Completed).

### Conclusion and Discussion

**Figure 3:**
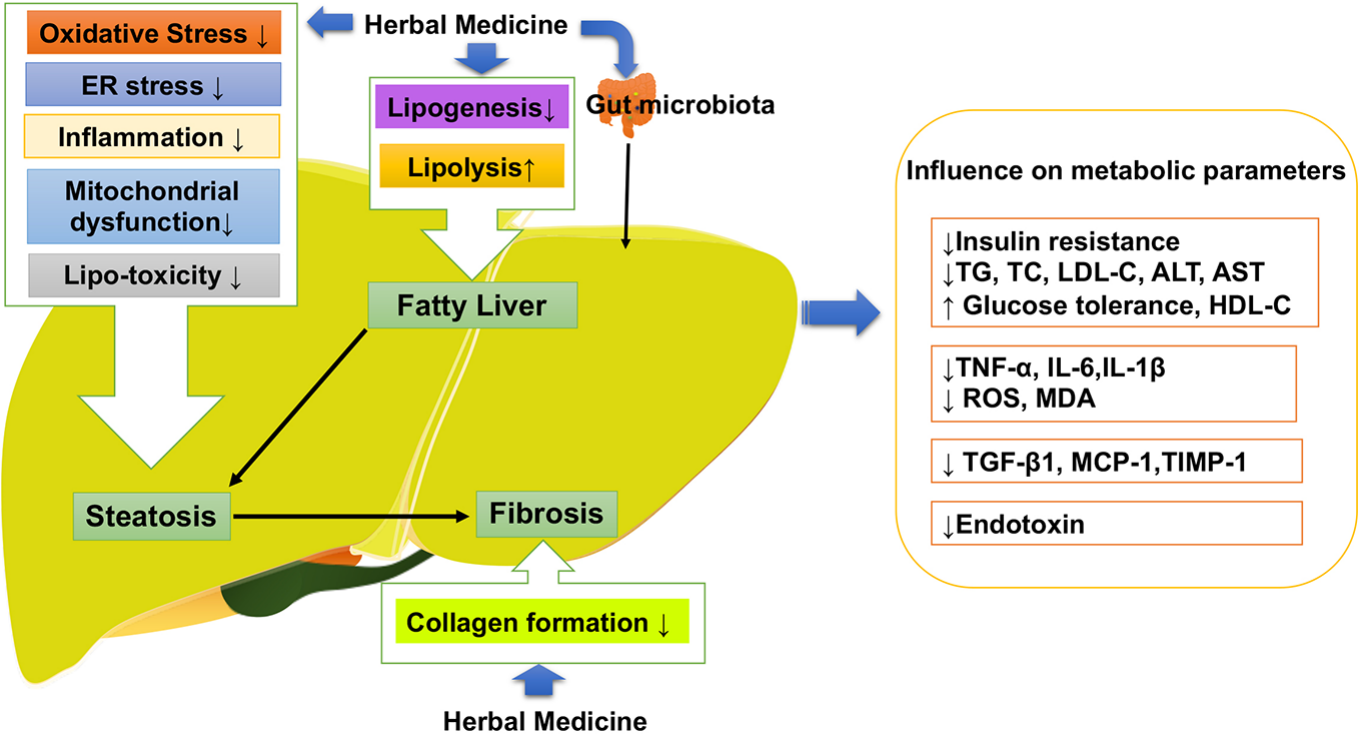
Herbal medicine mediated the key pathological events in the procession of NAFLD. NAFLD, Non-alcoholic fatty liver disease.

Following the general requirement of the pharmacological research of herbal medicine (Heinrich et al., 2020), we firstly made assessment of the pharmacological research literature on herbal medicine, focusing on the experiment design, we checked the methodological details such as group size, controls and animal species. Specifically, we observed the dosage, route and frequency of drug administration in the research literature, and checked the tested dose range that should be pharmacological relevant. After the assessment, the literature of herbal medicine that reach the general requirement of the pharmacological research are included in our review. As shown in **Table 1**, a large number of natural compounds, whole extract and herb formula have been widely investigated against different pathologies of NAFLD with promising results (Porras et al., 2018). Increasing evidence has shown polyphenols (Porras et al., 2018) including resveratrol (Aguirre et al., 2014), quercetin (green tea, soy isoflavones) silymarin (extracted from *Sylbum marianum*), silybin and rutin (Van De Wier et al., 2017) are the frequently investigated natural compounds, along with the satisfactory effectiveness in NAFLD. As shown in **Figure 3**, herbal medicine mediated the key pathological events in the procession of NAFLD include lipid metabolism dysfunction, insulin resistance, fibrosis, oxidative stress, inflammation, and apoptosis (Wang et al., 2016). The satisfactory improvement of NAFLD disease outcomes and endpoints mentioned the amelioration or reduction of fat mass, insulin resistance, serum level of FFA, AST, and ALT, hepatic lipid accumulation and fibrosis, as well as hepatic oxidative stress, inflammatory response, and apoptosis. Thus, we made a review of the usage and role of herbal medicine in NAFLD. Steatosis, characterized by fat accumulation, represented the early stage of NAFLD and inflammation that interfered with the insulin signaling pathway is the key process that makes early steatosis develop into steatohepatitis (Bai and Li, 2019). Therefore, amelioration of steatosis and inflammation is vital for NAFLD therapy. Herbal medicine therapy has shown promising anti-inflammatory, antioxidant, and anti-apoptotic properties that might take beneficial effects on curtailing the inflammatory progression of NAFLD. Their action was always involved in multi-pathways to improved NAFLD, such as baicalin exerted anti-inflammation and anti-oxidant effects that can reduce hepatic lipid accumulation, suppress induced hepatic inflammation, and prevent liver fibrosis involved inhibiting hepatocyte apoptosis (Zhang J. et al., 2018). Gegenqinlian decoction influence NAFLD *via* improving PPARγ to inhibit inflammation and modulated lipid metabolism (Wang et al., 2015). *Alisma orientalis* protected against *de novo* lipogenesis to upregulate hepatic lipid export. Additionally, it modulated oxidative stress cytokines, inflammatory and fibrotic mediators, eventually influenced lipo-apoptosis and liver injury panels (Choi E. et al., 2019). Resveratrol can be considered as a pharmacological SIRT1 activator (Zhang et al., 2015) and take effects on hepatic steatosis by improving lipid-related gene transcriptional expression, oxidative stress and inflammation (Cheng et al., 2019), meanwhile decreasing ER stress (Ding et al., 2017) *via* the autophagy (Liu et al., 2015a). In addition, it has shown that herbal medicine combined with other interventions exhibited a much better beneficial effect than single interference alone. For example, Lingguizhugan decoction and calorie-restriction therapy together could enhance the reduction of fasting blood lipid levels (Yuanyuan et al., 2015; Cao et al., 2016). The combination of Korean red ginseng and probiotic *Lactobacillus* synergistically ameliorated hepatic inflammation (Kim et al., 2019a). The combination of Ganmaidazao and Shengmai-Yin decoction is applied as adjuvant therapy for Type II diabetes mellitus *via* activation of HSL, PPARα, and AMPK/PI3K/AKT, and inhibition of SREBP-1/FAS, influencing insulin sensitivity, and lipid biosynthesis (Li et al., 2019). Two types of anti-dyslipidemia herb formulas (Fenofibrate and xuezhikang) have been simultaneously applied in the treatment of NAFLD (Hong et al., 2007) as well as combined use of *Fructus Schisandrae* with statin showed anti-oxidative effect and inhibitory effect against liver toxicity (Wat et al., 2016). *Schisandra chinensis* Baill has been used as a complementary therapy for rosiglitazone and alleviated NASH with significantly lower levels of LDLC and SOD in liver than rosiglitazone (Yao et al., 2014; Wat et al., 2016). Therefore, herbal medicine supplement combined with other therapeutic approaches might provide feasible therapeutic strategies for patients with NAFLD (Porras et al., 2018).

**Table 1:** The recent research for herb medicine in the setting of NAFLD therapy.

Herbal Medicine	Source organism	Pharmacological model	Treatment (Pharmacological model and Duration)	Effective Dosage	Reported mechanism of action		
					Lipogenesis	Modulation of inflammatory parameters	others
S-allylmercaptocysteine	Garlic-derived	HFD induced obese rat	i.p, 3 times/week	200 mg/kg	↓	↓NF-κB and AP-1	↓Collagen formation ↓Oxidative stress ↑Autophagy
Dioscin	Polygonati Rhizoma	HFD induced obese rats	Orally,	60 mg/kg		↓ IκBα, p50 and p65	↓Collagen formation
		HFD induced obese Wistar rats and mice	Orally, 8 weeks	15–80mg/kg	↑SIRT1/AMPK		↓**Apoptosis**
		Dimethylnitrosamine-induced acute liver injury mice	Orally,	80 mg/kg	↓LXRα	↓	↓**Oxidative stress(**↑SIRT1, Nrf2)
Resveratrol		MCD diet induced NAFLD mice	Orally,	100 or 250 mg/kg/day		↓TBARS	↑**Autophagy**
		AML12 cells		25, 50, or 100 μmol/L	↓ FFA uptake.		
		C57BL/6J mice and as ULK1+/− mice with HFD	Orally, 4 weeks.	50 mg/kg		↓ IκBα-NF-κB	↓ **Oxidative stress**
							↓Fibrosis
		Mice with HFD for 4 weeks	Orally, 4 weeks.	0.40%			↓**Oxidative stress**
		HepG2 cells	24 h	20, 40, and 80μM			
		Wistar rats with HFD	Orally, 18 weeks	200 mg/kg		↑ cAMP-PRKA-AMPK-SIRT1	↓ ER stress(↑SIRT1)
Naringenin	Citrus-derived flavonoid	LDLR−/− mice with HFD	Orally, 4 weeks	1% or 3% wt/wt	↓ VLDL		↑ insulin resistance
Yangonin	Piper methysticum	Mouse fed with HFD	Orally, 16 weeks	10, 20, or 40 mg/kg	↓SREBP-1c pathway;↑ fatty acid β-oxidation		↑insulin sensitivity
							↓Fibrosis (↑ FXR)
Berberine	Coptidis Rhizoma	Mice fed with HFD	Orally; for 4 weeks	300 mg/kg/day	↓ SCD1 *via* AMPK-SREBP-1c pathway	p38MAPK/ERK-COX2 pathways	↓Fibrosis
		AML12		20 μM			
		NASH-HCC mice model	Orally, 12 week	250 mg/kg			
Betulinic acid	Outer bark of tree species	Mice fed with HFD	Orally, 11 weeks	50 mg/kg,	↓ SREBP-1c, ApoC2, RBP4, FAS, and SCD- 1; ↑AMPK ↑ fatty acid oxidation	↓F4/80, IL-1α, IL-1β, IL-6, TNF-α	↓Fibrosis (↑ FXR)
		AML12 treated with palmitic acid (PA)		50 μg/ml			
		Mice fed with MCD and HFD	Orally, 6 weeks	100 mg/100 g diet			
Glycyrrhizin	Glycyrrhizae Radix Et Rhizoma	Mice fed with MCD diet	i.p, 2 weeks	50 mg/kg per day	↓ lipogensis	↓NLRP3	
						↑FXR	
Gastrodin	*Gastrodia elata* Bl	Larval zebrafish fed with HFD		10, 25, 50 mg/L	↓ lipogensis	↓TNFα, IL-6, and IL1β	↓Fibrosis ↓TGFβ1)
							**Oxidative stress** ↑NRF2, HO-1
Naringenin	Citrus-derived flavonoid	*Ldlr*−/− mice fed a Western diet	Orally, 4 weeks	1 or 3%	↓ SREBP1c; ↓VLDL;↑ fatty acid oxidation		↓ hyperinsulinemia
Puerarin	*Pueraria lobate* (Willd.)Ohwi	Mice fed with a high-fat +high-sucrose diet	Orally, 18 weeks	0.2,0.4g/kg/day	↓liver steatosis	↓	↓Fibrosis ↓TGFβ1)
Silibinin	*Silybum martanum* (L.) Gaertn.	MCD diet induced NASH mice	Orally, 6 weeks	10 and 20 mg/kg/day	↑ β-oxidation	↓NASH *via* CFLAR-JNK pathway,	**Oxidative stress** ↑CAT, GSH-Px and HO-1; ↓CYP2E1, CYP4A
		NCTC-1469 cells treated with OA plus PA		50 and 100 μmol/L			
		HepG2 cells treated with OA		5, 20, 50, and 100 μM	↓PPARα, SREBP-1C and PNPLA3	↓NO	↓glucose uptake (PI3K-AKT) ↓**oxidative stress** (NRF2, CYP2E1, CYP4A)
Sparstolonin B	*Sparganium stoloniferum* Buch-Ham	High-fat-fed mice	ip, for 4 week	3 mg/kg, twice a week	↓TLR4 lipid raft trafficking	↓ TLR4 pathway	↓Fibrosis ↓TGFβ1)
							↓NADPH oxidase activation.
		Kupffer cell line		100 μg/ml			
		LX2 cells with LPS (100 ng/ml)		10, 100 μM			
Isochlorogenic acid B	Laggera alata (Asteraceae)	Mice fed with MCD diet	Orally, 4 weeks	5, 10 and 20 mg/kg.			↓Fibrosis (↓TGFβ1, LXO,MCP-1, COL1α1 and TIMP-1.)
							↓ oxidative stress (↑Nrf2)
Swertiamarin	Swertia bimaculata	Fructose-fed mice for 12 week	Orally, 4 weeks	25, 50 and 100 mg/kg	↓ SREBP-1/FAS/ACC	↓hepatic pro-inflammation	↓ hepatic xanthine oxidase (XO) ↑Nrf2
Baicalin	*Scutellaria baicalensis* Georgi	MCD diet-induced NASH	Orally, 4weeks.	50 and 100,200 mg/kg	↓	↓ inflammation	↓Fibrosis ↓ hepatic apoptosis.
Ursolic acid(UA)		Mice fed with HFD	Orally, 16 week	0.05% (w/w) UA diet			↓Fibrosis
		T090-induced mouse model.	Orally, 7 days	100, 250 mg/kg/day	Liver X Receptor α antagonist		
		L02 cells treated with PA		10–30 μg/ml	↓ lipid accumulation		↓oxidative stress.
		Rat fed with HFD	Orally, 6 weeks.	0.125%, 0.25%, 0.5% UA diet		↓ inflammation	↓ insulin resistance
Andrographolide	*Andrographis paniculata* (Burm. f.)Nee	Mice fed with CDAA diet	i.p, 22 weeks	1 mg/kg, 3 times/week		↓hepatic inflammation	↓collagen formation
		Fat-laden HepG2 cells.		50 μM		↓ NF-κB	
Ginsenoside Rb1	*Panax Ginseng* C.A.Mey	Rat fed with HFD	ip	10 mg/kg	↑CPT1		
		db/db mice	i.p, 14 days.	20 mg/kg	↓	↓	
Nobiletin	*Citrus reticulata* Blanco	Mice fed a high-fat diet	Orally, 16 weeks.	0.02%, w/w		↓ NLRP3	
		High glucose induced hepG2 cells		5, 25, and 50 μM	↑AMPK		
Ginsenoside Rb2	*Panax Ginseng* C.A.Mey	HepG2 cells		50 µmol/L			↑Autophagy
		db/db mice	i.p, 4 weeks	10 mg/kg	↑AMPK or SIRT1		
Akebia saponin D	*Dipsacus asper* Wall.ex Henry	ob/ob mice fed with HFD	i.p, 4 weeks	30,60,120 mg/kg,			**↑Autophagy**
		OA stressed Buffalo rat liver cells		1, 10, and 100 μM			↑ LC3-II ↓P62
Glycycoumarin	Rhizoma Glycyrrhizae	PA stressed cells (HepG2, AML-12, and L02)		10–40μM			↓Mitochondrial apoptosis(↓GSK-3,↓JNK)
		MCD diet induced NASH mice	i.p. 4 weeks	GCM 15 mg/kg/day	↓Lipogenesis	↓Inflammation	↓ Fibrosis; Oxidative stress
ethanol extract	*Lycopus lucidus* Turcz. ex Benth	HepG2 cells treated with OA plus PA		250–1000 mg/ml.	↑PPARα, AMPK ↓SREBP-1c		
		Mice fed with HFD	Orally,14 weeks.	100 or 200 mg/kg/day		↓TNF-α	
Danshen aqueous extract	*Salviae miltiorrhiza* Bge.	Mice fed with ethanol	Orally, 9 days	0.093, 0.28, 0.84 g/kg	↓Lipogenesis	↓Inflammation	↓ Fibrosis
Jwa Kum Whan	Scutellariae Radix and Euodiae Fructus	Mice fed with HFD	Orally, 15 week	100,200 mg/kg daily			↑Insulin Signaling
		HepG2 cells		10, 25, 50, 75, or 100 μg/ml			
Ethanol Extract	*Leonurus japonicus* Houtt.	Mice fed with HFD	Orally, 14 weeks	100 or 200 mg/kg	↑ AMPK, PPARα		↑Insulin Signaling
		1 mM free fatty acid induced HepG2 cells		250, 500, 750, or 1000 μg/ml	↑ AMPK, PPARα		
honeyberry extract	*Lonicera caerulea*	Mice fed with HFD	Orally, 6 weeks.	0.5%, 1%	↑ AMPK, CPT-1,PPARα		
polysaccharides	*Lycium barbarum* L.	Rat treated with HFD	Orally, 4 weeks	1 mg/kg,	↓lipid accumulation ↑ fatty acid oxidation		
methanolic extract	*Alisma orientale* (Sam.)Juzep.	Rats fed with HFD for 6 weeks	Orally, 6 week	150, 300, and 600 mg/kg	↑AMPK, PPARα	↓Inflammation	↓apoptosis;↓ oxidative stress;↑insulin sensitive
Seed Extract	*Psoralea corylifolia L*.	Mice fed a HFD	orally, 12 weeks	300 or 500 mg/g/d,	↓Lipogenesis	↓Inflammation	↑Insulin Signaling
Extract	*Schisandra chinensis* (Turcz)Baill	Wister rats fed with HFD	56 days	100 mg/kg/day	↓ LDLC		↓Endoplasmic reticulum (ER) stress
Total saponins	*Aralia elata* Seem.	ApoE-/- mouse fed with HFD.	i.g., 12 weeks.	75, 150 mg/kg/day		↓Inflammation	**↓**oxidative stress.
Total alkaloids	*Rubus aleaefolius* Poir.	HFD for 8 weeks	orally, 4 weeks	1.44, 0.72 g/kg	↓FAS, ACC ↑CPT		
Polygonatum kingianum		Rat fed with HFD	Orally, 14 weeks	1, 2, 4g/kg			Remedy mitochondrial dysfunction
Ethyl acetate extract	*Aristolochia manshuriensis* Kom	HFD-induced NASH model	15 weeks	2.5 mg/kg		↓Inflammation	↓oxidative stress.
							↓apoptosis
Fructus Schisandrae		SD rats fed with HFD for 8 weeks:	orally, 8 weeks	0.45% FS+0.3% Atorvastatin			↓oxidative stress.
Aqueous extract	*Salvia miltiorrhiza* Bunge	Ovariectomized (OVX)+ hyperlipidemic SD rats	Orally, 12 weeks	600 mg/kg/d			↓ Fibrosis
The chloroform extract	*Cyclocarya paliurus*	SD rats fed with HFD for 6 weeks	Orally, 4 weeks.				↓ Fibrosis
Saponins Raw and processed	*Panax Notoginseng* (Burk.) F.H.Chen	*CCl*_4_ induced fibrosis in rat	i.p	130 mg/kg		↓Inflammation	↓ Fibrosis
Citrus aurantium Peel Extract	*Citrus aurantium* L. *(Rutaceae*)	Mice fed with HFD	orally, 8 weeks	50, 100 mg/kg	↓PPAR-γ, SREBP-1c	↓ inflammation	
Korea red ginseng	*Panax ginseng* C.A.Mey	Fatty Rats	orally, 2 months	200 mg/kg/day		↓ inflammation	↓oxidative stress.
*Celastrus orbiculatus* Thunb.		ApoE(-/-) mice	Orally, 6 weeks	10.0 g/kg/d	↑adiponectin	↓TLR4 and NF-κB p65, TNF-α.	
		A guinea pig of NAFLD	Orally, 8 weeks		↑CYP7A1 and HMGCR		↓NO and iNOS levels
BaiHuJia RenShen Decoction	Anemarrhenae Rhizoma, Gypsum Fibrosum, Glycyrrhizae Radix Et Rhizoma,Ginseng Radix Et Rhizoma	HuS-E/2 cell with PA			↑P-AMPK;P-ACC; ↓SCD1 ↑CPT		
		db/db mice	orally, 6 weeks	900 mg/kg			
BuShenKangShuai tablet		ApoE (-/-) mice	Gavage, 6 weeks	BSKS or atorvastatin		↑adiponectin ↓TLR4 and NF-κB p65	
LiGanShiLiuBaWei San	Punica granatum, Cinnamomum cassia, Elettaria cardamomum, Piper longum, Carthamus tinctorius, Amomum tsao-ko	Rat with HFD	Orally, 4 weeks	0.75 and 1.5 g/kg	↑PPARα		↓iNOS levels
		HepG2 with FFAs			↑PPARβ		
Hugan Qingzhi tablet	Alismatis Rhizoma, Crataegi Fructus, Typhae Pollen, Nelumbinis Folium, Notoginseng Radix Et Rhizoma	L02 and HepG2 cells induced by FFA				↑SIRT1 ↓Ac-NF-κB-p65	
		Rat with HFD	Orally, 12 weeks	2.16/1.08/0.54 g/kg			
Sinai san decoction	Bupleuri Radix, Paeoniae Radix, Aurantii Fructus Immaturus, Glycyrrhizae Radix Et Rhizoma	Rat with HFD+CCL4	Orally, 8 weeks	0.1 ml/kg/day		↓ inflammation	
Gegenqinlian Decoction	PuerariaeLobataeRadix, Coptidis Rhizoma, Scutellariae Radix, Glycyrrhizae Radix Et Rhizoma	Rat with HFD	Orally, 8 weeks	5.04, 10.08 g/kg/day	↓PPARγ		
Tangzhiqing Decoction	Mori Folium, Nelumbinis, Crataegi Folium, Salviae Miltiorrhizae Radix Et Rhizoma, Paeoniae Radix Rubra	Rat with HFD	Orally, 4 weeks	540 mg/kg/d	↓steatosis		
Qushi Huayu Decoction	Artemisiae scopaiae Herba, Polygoni cuspidati Rhizoma Et Radix, Hyperici Japonici Herba, Curcumae longae Rhizoma, Gardenia jasminoides Ellis	Rat with HFD	Orally, 4 weeks	0.1 ml/kg·d,	↑AMPK and ACC		
		L02 cells		5%–15% QHD serum	↓ cellular TG		
Hugan Qingzhi tablet	Alismatis Rhizoma, Crataegi Fructus, Typhae Pollen, Nelumbinis Folium, Notoginseng Radix Et Rhizoma	Rat with HFD	Orally,	10% HQT-medicated serum	↓	↓IL-6, ↓P65	
				90 mg/kg, combination with calorie restriction			
Lingguizhugan decoction	Poria, Ramulus Cinnamomi, Atractylodis Macrocephalae Rhizoma,Glycyrrhizae Radix Et Rhizoma	Mice with HFD	16 weeks	Fecal microbiota transplantation	↓		
Tangshen formula	Puerariae Radix, Astragalus, Ligustrum lucidum Ait, Ganoderma, Salvia miltiorrhizae Radix Et Rhizoma, Rhei Radix Et Rhizoma	Mice with HFD	Orally, 16 weeks	2.4 g/kg/day	↑AMPK/SIRT1		
		HepG2 cells		25, 50, 100 μg/ml			
Fenofibrate and Xuezhikang		Rat fed with HFD	Orally, 6 weeks	F (100 mg/kg) and X (300 mg/kg)	↑PPARα	↓TNF-α.	
Bangpungtongseong-san	Talcum, Glycyrrhiza uralensis,Gypsum, Scutellaria baicalensis, Platycodon grandiflorum, Ledebouriella seseloides, Cnidium officinale, Angelica gigas,Paeonia lactiflora,Rheum undulatum, Ephedra sinica, Mentha pulegium, Forsythia koreana, Erigeron canadensis, Schizonepeta tenuifolia, Atractylodes japonica, Gardenia jasminoides, Zingiber officinale	HFD induced obese mice	12 weeks	1.5% w/w	↑ mitochondrial function		antioxidant

## Author Contributions

YF and designed and conceived the study. YX, NW, and YF retrieved, analyzed the data and drafted the manuscript. WG, CZ, FC, HT, SL, and NW discussed and revised the manuscript. All authors confirmed final version of the manuscript.

## Funding

This research was partially supported by the Research Council of the University of Hong Kong (project codes: 104004092 and 104004460), Wong’s donation (project code: 200006276), a donation from the Gaia Family Trust of New Zealand (project code: 200007008), the Research Grants Committee (RGC) of Hong Kong, HKSAR (Project Codes: 740608, 766211, 17152116, and 17121419), Health and Medical Research Fund (Project code: 15162961, 16171511, and 16172751).

## Conflict of Interest

The authors declare that the research was conducted in the absence of any commercial or financial relationships that could be construed as a potential conflict of interest.
